# Relation between primary and secondary health care coverage with periodontal disease in Brazil: a multilevel study

**DOI:** 10.1590/1980-549720260008.supl.1

**Published:** 2026-06-12

**Authors:** Roger Keller Celeste, Leonardo Vilar Filgueiras, Joao Paulo Steffens, Stefany Duarte dos Anjos, Rafael Aiello Bomfim, Yuri Wanderley Cavalcanti, Alex Nogueira Haas

**Affiliations:** I Universidade Federal do Rio Grande do Sul – Porto Alegre (RS), Brazil.; II Universidade Federal do Paraná – Curitiba (PR), Brazil.; III Universidade Federal do Mato Grosso do Sul – Campo Grande (MS), Brazil.; IV Universidade Federal da Paraíba – João Pessoa (PB), Brazil.

**Keywords:** Dental health services, Secondary health care, Primary health care, Periodontitis, Specialties, dental, Health services evaluation

## Abstract

**Objective:**

To evaluate the independent and combined effects of the presence of Dental Specialty Centers (*Centros de Especialidades Odontológicas* – CEOs) and primary health care coverage on Periodontal Disease (PD) among adults and older adults in Brazil.

**Methods:**

Data from the SB Brasil 2023 survey were obtained for 7,851 individuals who used public services. Contextual variables were obtained from health information systems and the Brazilian Institute of Geography and Statistics (*Instituto Brasileiro de Geografia e Estatística* – IBGE). The outcome variable was PD, defined as having >1 tooth with probing depth >3 mm. The main exposures were the presence of CEOs and the coverage of Primary Oral Health Care (PHC-OH). Multilevel logistic regression analyses were performed.

**Results:**

The weighted prevalence of PD was 17.4%; and, respectively, 19.4% and 11.9% (p=0.03) in municipalities with and without CEOs. Municipalities with PHC-OH population coverage of ≤50, 50–74, 75–99%, and 100% showed prevalences of 19.3, 21.1, 18.1, and 7.7%, respectively (p=0.01). Individuals in municipalities with CEOs had higher odds of developing PD [OR 1.12 (95% CI 0.75–1.68)]. Individuals in municipalities with 100% PHC-OH coverage had OR 0.57 (95% CI 0.29–1.12), indicating lower odds of PD compared to municipalities with <50% coverage in the presence of CEOs. In the absence of CEOs, the corresponding OR was 0.43 (95%CI 0.19–0.97), but the interaction between CEO and PHC-OH was not significant (p=0.90).

**Conclusion:**

Greater coverage of primary oral health care appears to be independently associated with a lower prevalence of PD in small- and medium-sized cities. Future studies should investigate barriers in the referral system.

## INTRODUCTION

The most prevalent periodontal diseases (PD), gingivitis and periodontitis, are characterized by infectious-inflammatory processes that, if left untreated, can result in masticatory dysfunction due to the destruction of the teeth-supporting structures^1,2^. Beyond their effects on mastication and aesthetics, periodontitis is associated with significant systemic consequences, including an increased risk of cardiovascular disease, diabetes, and pregnancy complications, highlighting its relevance as a public health concern^3^. Moreover, it disproportionately affects individuals across different social strata^4^ and exerts a substantial impact on quality of life^5^.

Clinically, periodontitis is defined by the presence of clinical attachment loss (CAL) and/or radiographic bone loss. Other inflammatory clinical parameters, such as probing depth (PD) and bleeding on probing (BOP), can complement the clinical diagnosis^6^. At the epidemiological level, the Community Periodontal Index (CPI) has been recommended by the World Health Organization (WHO) for its ease of application in large populations; however, it is limited by its partial assessment of the dentition, examining only six index teeth, and by not evaluating CAL. Approximately 12% of the global population is affected by the disease in its most severe form^7^, while at least 80% of adolescents present signs of gingivitis^8,9^. In Brazil, data from the 2023 National Oral Health Survey (SB Brasil 2023)^10^ indicate that 13.4% of adults aged 35 to 44 years exhibit periodontal pockets, representing the age group with the highest prevalence of the disease.

Approximately 80% of Primary Health Care Units (*Unidades Básicas de Saúde* – UBS) include dentists in their teams^11^. Despite substantial advances in funding and the expansion of the oral health care network resulting from the National Oral Health Policy^12^, various challenges persist, including the organization of demand and the regionalization of care within the network^13,14^. Service utilization and access inequalities are generally influenced by socioeconomic and organizational factors; although overall utilization has increased, disparities in the use of dental services remain^15^. Furthermore, difficulties in aligning resources and public health policies with population needs hinder the equitable distribution and use of oral health services^16,17^.

Evidence regarding the effects of primary care service utilization on oral health, as well as the role of secondary care in dental treatment, remains limited^18–20^. A recent systematic review indicates that some studies report a modest impact of primary care use on periodontal health, with greater coverage being associated with a lower prevalence of PD^21^. However, most studies do not consider specialized dental care, which in Brazil has been expanded through Dental Specialty Centers (*Centros de Especialidades Odontológicas* – CEOs).

The presence of CEOs appears to be associated with a lower prevalence of PD among adults, with this effect partially mediated by service utilization^19^. Consequently, expanding access may increase the use of services and potentially improve population-level oral health. Historically, investments have prioritized primary oral health care; however, specialized services, including periodontics, have recently been expanded through CEO funding in Brazil^12,22^. Although this policy has led to increased utilization of specialized services, its epidemiological impact remains unclear.

Expanding access to specialized services may enhance the effectiveness of Primary Health Care (PHC) and, consequently, influence the epidemiological profile of oral health. For instance, CEOs have been reported to reduce the proportion of tooth extractions in municipalities with greater coverage of oral health teams within the Family Health Strategy^23^. The effectiveness of PHC can be further improved, even at constant coverage levels, if cases requiring higher technological capacity are referred to network points equipped to address such needs. This approach allows PHC teams to focus on demand in accordance with their capabilities. The present study hypothesizes that PHC teams are better able to improve the epidemiological profile of oral health in municipalities with CEOs. Accordingly, the objectives are to evaluate the independent and combined effects of the presence of CEOs and PHC coverage on PD among adults and older adults in Brazil.

## METHODS

This cross-sectional study utilized data from multiple sources. Individual-level variables were obtained from the 2023 SB Brasil database^24^, while contextual variables were collected from the Health Information Systems (*Sistemas de Informação em Saúde* – SIS) of all 430 municipalities included in SB Brasil. The original sample comprised 18,766 individuals aged 35 to 44 and 65 to 74 years. The present study focused on individuals who reported using public services in their most recent dental consultation, resulting in a sample of 7,851 eligible participants. This criterion was applied because public services are unlikely to influence users of other types of dental care.

### Outcome Variable

The outcome variable was the presence of PD (dichotomous: 0=no, 1=yes), measured at the individual level. Six index teeth, one per sextant, were examined using the CPI with a WHO probe. Only sextants containing two or more teeth without indication for extraction were evaluated. Each sextant was assigned a score based on the presence (codes 3 and 4) or absence (codes 0, 1, and 2) of PD>3 mm. The highest score among all sextants was used to represent the individual. PD was defined as having ≥1 sextant with PD>3 mm (CPI codes 3 or 4).

### Exposure variables

All exposure variables were measured at the municipal level. The primary exposure variables were the presence of a CEO (none, 1 or more) and oral health coverage in primary care (≤50, 50–74, 75–99, or 100%). For the variable "presence of a CEO," this dichotomization was justified by the small number of municipalities with multiple CEOs in the sample (45% none, 41% with 1, 7% with 2, and 7% with more than 2 centers). Regarding oral health coverage in primary care, the categorization was based on its distribution, avoiding simple dichotomization, and considering that 33% of municipalities in the sample have full (100%) coverage, which includes 10.8% of individuals in the SB Brasil database. Consequently, the coverage categories (<50, 50–75, 75–99, and 100%) were established to balance the number of municipalities and individuals within each category, ensuring a more equitable sample distribution.

The number of CEOs in each municipality was obtained from the Ministry of Health (General Coordination of Oral Health), available at: https://www.gov.br/saude/pt-br/composicao/saps/brasil-sorridente/cidades-atendidas/ceo. Data were extracted from the Ministry of Health's e-Gestor system, using December 2022 as the reference month, and are available at: https://relatorioaps.saude.gov.br/cobertura/saude-bucal/v2. Oral health coverage was calculated based on total coverage, including oral health teams in the Family Health Strategy (e-SFSB teams) as well as those in Primary Health Care (PHC-OH).

In addition to these variables, the number of referrals for periodontal treatment was obtained from the Primary Care Information System (*Sistema de Informação da Atenção Básica* – Sisab), along with the total number of periodontal intervention procedures (including supra- and subgingival scaling) performed in primary care between January/2022 and December/2023. A rate was subsequently calculated by dividing the number of referrals by the total number of scaling procedures.

### Adjustment variables

**Figure 1:**
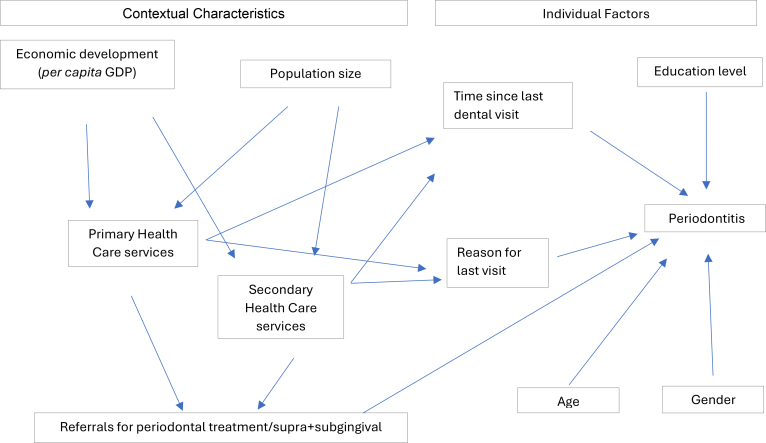
Theoretical-operational model of analysis.

Using a theoretical-operational model developed for this study (Figure 1), the primary associations were adjusted for potential confounding factors. The model also enabled the identification of potential mediating factors in the relationship between the main exposures and the outcome. Covariates included in the model were selected based on previous studies described in the Introduction and were grouped as individual- or contextual-level confounding or mediating factors. Unlike the theoretical-operational model, household income was excluded due to a high proportion of missing values (26.5%, n=2,084).

Individual-level variables for compositional control, obtained from the SB Brasil survey, included gender (male/female), age (years), education level (incomplete primary, complete primary, complete secondary, and complete higher education), time since the last dental visit (<1 year, 1 to 2 years, 2 to 3 years, >3 years), and reason for the visit (check-up/maintenance, pain/extraction, treatment). Municipal-level variables, such as population size and Gross Domestic Product (GDP) in reais, were obtained from the 2022 Census via the Brazilian Institute of Geography and Statistics (*Instituto Brasileiro de Geografia e Estatística* – IBGE) website. GDP per capita was calculated and categorized into tertiles, with the first tertile representing the lowest monetary values. Population size was classified into four categories (up to 100,000 inhabitants, 100,000–500,000 inhabitants, 500,000–1,000,000 inhabitants, and >1,000,000 inhabitants).

### Statistical analysis

Descriptive data were weighted to estimate prevalences and representative means. Sample weights were calculated based on the sampling fractions at each stage and calibrated to adjust for non-response. Multilevel logistic regression with a random intercept was used to estimate the adjusted effects of the primary municipal variables on PD, with individuals nested within their respective municipalities as second-level units. A logit link function was applied, and exponentiated β coefficients are presented as odds ratios (ORs). Finally, the Variance Partitioning Coefficient (VPC) was calculated using the D method proposed by Goldstein et al.^25^

Initially, interaction models were fitted for confounding factors according to the theoretical-operational model (Figure 1), using a hierarchical analytical structure. Subsequently, four multiple regression models without interactions were estimated, in addition to models assessing unadjusted effects. The first model (M1) examined the association between primary care coverage and the outcome, adjusted for confounding factors at the individual level. The second model (M2) assessed the association between CEO presence and the outcome, similarly adjusted for individual-level confounders. The third model (M3) included all individual- and contextual-level variables, whereas the fourth model (M4) excluded health service utilization variables (potential mediators). Additional analyses using bivariate tables for categorical variables identified strong collinearity (zeroed cells or small sample sizes) between CEO presence and PHC-OH coverage with population size; therefore, a stratified analysis was conducted. Model fit was evaluated using the Bayesian information criterion. All analyses were performed using Stata version 19.0.

#### Data availability statement:

Data are available upon request. The complete dataset supporting the findings of this study can be obtained from the Ministry of Health (https://www.gov.br/saude/pt-br/composicao/saps/brasil-sorridente/sb-brasil/dados) upon request.

## RESULTS

The study sample comprised 7,851 eligible individuals. Of this total, 18.7% (n=1,465) were excluded because 16.3% (n=1,273) were edentulous and 2.4% (n=192) had missing data on PD, resulting in a final analytical sample of 6,386 individuals. The variance partition coefficient indicated that 17.1% of the total variability was attributable to differences between municipalities; after adjustment for compositional confounding, this proportion remained at approximately 14% (Supplementary Table 1).

The overall prevalence of PD was 17.4% (Table 1). Prevalence was significantly higher in municipalities with a CEO (19.4%) than in those without such centers (11.9%). Higher prevalences were also observed in municipalities with lower PHC-OH coverage. No statistically significant differences in PD prevalence were observed across categories of referrals for periodontal treatment. Higher prevalences were identified in municipalities with higher GDP and larger population sizes; however, these differences were not statistically significant (p<0.05). Regarding individual-level variables, no statistically significant differences were observed by gender or income. However, a higher prevalence was found among older adults compared with adults, as well as among individuals with lower levels of education compared with higher education. With respect to time since the last dental visit, individuals who had visited the dentist 2 to 3 years had a higher prevalence of PD. Similarly, higher prevalence was observed among individuals who sought emergency care compared with those who sought care for health maintenance.

**Table 1:** Weighted prevalence of periodontal disease (CPI≥3) in adults and older adults using public services according to municipal contextual and individual variables in Brazil, 2023.

		Total		Weighted prevalence		
		%	n	%	Cases	p-value
Total		100.0	6,386	17.4	1,254	
Contextual (municipal) variables						
Presence of CEO						
	None	26.7	827	11.9	827	0.03
	1 or more	73.3	5,559	19.4	5,559	
Coverage of primary oral health care services						
	<50%	46.7	4,185	19.3	3,487	0.01
	50–74%	21.2	1,381	21.1	1,146	
	75–99%	12.8	1,385	18.1	1,092	
	100%	19.4	900	7.7	661	
Referrals for periodontal treatment / supra+subgingival scaling						
	1^st^ tertile (lowest)	44.4	1,991	15.3	1,991	0.43
	2^nd^ tertile (intermediate)	30.3	2,192	20.3	2,192	
	3^rd^ tertile (highest)	25.4	2,203	17.6	2,203	
GDP per capita						
	1^st^ tertile (lowest GDP)	41.0	1,999	14.1	1,999	0.09
	2^nd^ tertile (intermediate GDP)	18.6	2,252	16.2	2,158	
	3^rd^ tertile (highest GDP)	40.5	2,135	21.0	2,229	
Population size (inhabitants)						
	Up to 100,000	34.9	1,059	13.8	1,074	0.18
	100,000–500,000	36.9	1,471	17.2	1,158	
	500,000–1 million	10.0	1,713	19.7	1,808	
	> 1 million	18.2	2,143	23.0	2,346	
Individual variables						
Gender						
	Male	32.5	2,065	19.9	2,065	0.13
	Female	67.5	4,321	16.2	4,321	
Age (years)						
	35 to 44	81.8	4,051	84.3	4,051	<0.01
	65 to 74	18.2	2,335	75.3	2,335	
Equivalent income (minimum wage)						
	≤1/2	22.7	927	16.0	927	0.34
	1/2–1	37.7	1,917	17.9	1,917	
	1–2	30.7	1,467	17.8	1,467	
	≥2	8.9	401	9.5	401	
Education						
	Incomplete elementary school	28.5	2,082	24.6	2,082	<0.01
	Complete elementary school	23.1	1,360	18.0	1,360	
	Complete high school	41.3	2,415	13.2	2,415	
	Complete Higher education	7.1	465	6.3	465	
Time since last dental visit (years)						
	Less than one	51.9	2,935	13.4	2,935	<0.01
	1 to 2	19.3	1,271	17.8	1,271	
	2 to 3	12.6	911	20.6	911	
	>3	16.3	1,269	27.2	1,269	
Reason for last visit						
	Check-up/maintenance	30.9	1,794	11.9	1,794	<0.01
	Pain/extraction	38.6	2,739	22.4	2,739	
	Treatment	30.5	1,840	16.4	1,840	

CEO: Dental Specialty Centers (Centros de Especialidades Odontológicas); GDP: Gross Domestic Product.

The prevalence of PD was examined according to the presence of a CEO (Supplementary Table 2) and the coverage of PHC-OH (Supplementary Table 3). A stronger association between PHC-OH and PD was observed only in municipalities without a CEO. The remaining municipal-level variables showed no significant association in either stratum defined by the presence or absence of a CEO. Regardless of PHC coverage, no differences in PD prevalence were observed across categories of the other municipal variables, although the pattern of association was similar. At the individual level, significant associations with periodontal disease prevalence were observed across certain categories of age, education level, time since the last dental visit, and reason for the dental visit.

Table 2 presents the interaction models between CEO and PHC-OH, which were not statistically significant in either the crude (p=0.88) or adjusted (p=0.90) analyses. In municipalities without a CEO, 100% PHC-OH coverage was associated with a 57% lower likelihood of PD (OR 0.43 95%CI 0.19–0.97). For the remaining OR estimates, the confidence intervals crossed the null value (OR=1). Additional interactions were tested with the presence of CEOs (CEO#population size, p=0.75; CEO#periodontal referrals, p=0.25; CEO#GDP, p=0.19; CEO#time since last dental visit, p=0.51; and CEO#reason for last dental visit, p=0.35) and with PHC-OH coverage (PHC#population size, p=0.11; PHC#periodontal referrals, p=0.31; PHC#GDP, p=0.79; PHC#time since last dental visit, p=0.14; and PHC#reason for last dental visit, p=0.61).

**Table 2:** Odds ratio and 95% confidence interval of having at least one tooth with a Community Periodontal Index (CPI) score ≥ 3 according to the presence of a Dental Specialty Center, stratified by levels of primary oral health care coverage in adults and older adults, SB Brasil 2023.

Number of CEOs	Coverage of primary oral health care services	Crude model		Adjusted model*	
		OR	(95%CI)	OR	(95%CI)
None	<50%	1		1	
	50–74%	0.98	(0.35–2.68)	0.88	(0.32–2.42)
	75–99%	0.84	(0.30–2.35)	0.68	(0.24–1.93)
	100%	0.46	(0.21–1.03)	0.43	(0.19–0.97)
At least one	<50%	1		1	
	50–74%	0.86	(0.57–1.31)	0.82	(0.55–1.24)
	75–99%	0.82	(0.50–1.36)	0.90	(0.54–1.51)
	100%	0.63	(0.34–1.15)	0.57	(0.29–1.12)
p-values for interaction between CEO and PHC coverage		0.88		0.90	

OR: odds ratio; CI: confidence interval; CEO: Dental Specialty Centers (*Centros de Especialidades Odontológicas*); GDP: Gross Domestic Product; PHC: Primary Health Care.

* Adjusted for: gender, age, education, time since last dental visit, reason for last visit, GDP per capita, and referral rate for scaling procedures in PHC.

Considering the collinearity between population size and the presence of CEOs and eSB in PHC, stratified models are presented in Table 3. The effect of total PHC-OH coverage was statistically significant only in municipalities with smaller population sizes, in which higher coverage was associated with a lower prevalence of PD (OR 0.41 95%CI 0.18–0.97). In larger municipalities, it was not possible to assess the association with increased coverage due to the absence of municipalities with >500,000 inhabitants presenting high coverage. A similar limitation was observed for CEOs, as all large municipalities had CEOs. Full models reporting ORs adjusted for population size and additional variables are presented in Supplementary Table 1. Notably, the association between 100% PHC-OH and lower prevalence of PD remained significant (OR 0.52 95%CI 0.31–0.89).

**Table 3:** Adjusted odds ratio with 95% confidence interval of having at least one tooth with a Community Periodontal Index (CPI) ≥3 in adults and older adults, stratified by population size, SB Brasil 2023.

		Model <100,000 inhab.		Model 100,000–499,000 inhab.		Model 500,000–999,000 inhab.		Model >1 million inhab.	
		OR	(95%CI)	OR	(95%CI)	OR	(95%CI)	OR	(95%CI)
Municipal variables									
CEO*									
	None	1		1		-	-	-	-
	1 or more	1.15	(0.50–2.66)	1.53	(0.63–3.70)	-	-	-	-
Primary oral health care coverage									
	<50%	1		1		1		1	
	50–74%	0.86	(0.31–2.39)	0.91	(0.42–1.97)	0.77	(0.40–1.48)	1.46	(1.18–1.80)
	75–99%	0.82	(0.32–2.10)	0.57	(0.24–1.33)	1.72	(0.25–11.6)	-	-
	100%	0.41	(0.18–0.97)	0.81	(0.30–2.15)	-	-	-	-
Referral tertiles†									
	T1 (lowest)	1		1		1		1	
	T2 (intermediate)	1.10	(0.40–3.01)	0.64	(0.31–1.31)	2.02	(0.48–8.42)	1.34	(0.94–1.92)
	T3 (highest)	0.79	(0.33–1.91)	0.81	(0.43–1.55)	2.83	(0.67–12.0)	1.20	(0.81–1.78)
GDP per capita tertiles‡									
	T1 GDP (lowest)	1		1		1		1	
	T2 GDP (intermediate)	0.68	(0.32–1.48)	0.69	(0.30–1.58)	0.76	(0.12–4.86)	0.77	(0.40–1.48)
	T3 GDP (highest)	1.20	(0.57–2.55)	0.83	(0.39–1.79)	1.33	(0.19–9.16)	1.58	(0.81–3.08)
Sample									
	Individuals	1,039		1,448		1,693		2,125	
	Municipalities	189		97		24		13	
Model fit									
	BIC	938.0		1,357.3		1,657.8		2,362.2	
	VPC	29.1%		17.5%		3.7%		2.1%	
	2nd-level variance	1.35		0.70		0.13		0.07	

inhab: inhabitants; CEO: Dental Specialty Centers (*Centros de Especialidades Odontológicas*); GDP: Gross Domestic Products.

Note: Models adjusted for age, gender, education, time since last dental visit, and reason for last visit.

* Presence of Dental Specialty Centers in the municipality;

† Referrals for periodontal treatment/supra+subgingival scaling in primary care;

‡ Gross Domestic Product per capita;

§ Model fit indicators.

## DISCUSSION

This study reports three main findings. First, no synergy was identified between the presence of CEOs and PHC-OH coverage. Evidence supporting such an interaction remains scarce, and no prior study has been identified, although the hypothesis is theoretically plausible^23^. Second, after adjustment, a non-significant association was observed between the presence of CEOs and the prevalence of PD, corroborating previous findings based on nationally representative data from Brazil in 2010^19^. Finally, a gradient was observed in the association between higher PHC-OH coverage and lower prevalence of PD, consistent with previous reports^21,26–28^. The fact that all municipalities with >500,000 inhabitants had CEOs, while none achieved 100% PHC coverage, suggests that these variables may reflect underlying urban structural characteristics (*e.g*., socioeconomic inequalities, urban mobility constraints, and health system overload), rather than a direct causal effect of service provision on PD. Accordingly, the association between PHC coverage and PD can only be inferred for municipalities with fewer than 500,000 inhabitants, given the lack of sufficient coverage in larger municipalities. The residual variance attributed to the municipal level, approximately 14%, indicates that contextual variables play a relevant role and warrant further investigation.

This study has both limitations and strengths. A limitation inherent to observational designs is the inability to establish a clear temporal relationship. Although service coverage represents a relatively stable municipal characteristic, urban mobility between municipalities with differing levels of coverage may be substantial, potentially introducing bias. Additionally, it was not possible to control for certain confounding variables, such as smoking and diabetes. Periodontitis is currently defined as a multifactorial chronic inflammatory disease, with diabetes and smoking recognized as classical risk factors that may even influence its classification in terms of progression risk (grade)^6^. More recently, an increasing body of evidence has characterized periodontitis as a comorbidity or component of multimorbidity with other noncommunicable chronic diseases (NCDs), generally arising from shared risk factors such as smoking^29^. Another important limitation concerns the use of CPI for defining periodontitis. The absence of information on clinical attachment loss from a full-mouth examination, considered the gold standard for periodontitis diagnosis, may have introduced misclassification bias in the disease definition within this SB Brasil sample.

The strengths of this study include the use of data from representative samples of the general population, restricted to users of public health services. In addition, official data from health information systems were employed. Furthermore, the outcome based on probing depth (periodontal pocket) is relatively sensitive for identifying treatment needs, in contrast to clinical attachment loss, which reflects the cumulative history of the disease. However, this methodological choice may have resulted in the inclusion of false-positive cases — such as individuals presenting gingival overgrowth due to different etiologies — or the exclusion of true cases of PD, particularly in progression phenotypes characterized by attachment loss without concomitant periodontal pocket formation^30^. Finally, it was not possible to estimate the extent and severity of periodontal disease due to methodological limitations, as the survey did not adopt a full-mouth examination protocol.

This study did not confirm an association between access to specialized dental services at the municipal level and PD. This absence of effect may be explained by several hypotheses. One possibility is that access to health services does not exert a substantial impact on the epidemiological profile. Previous studies have reported weak or absent effects of dental services on outcomes such as dental caries, dental calculus, periodontitis, and gingivitis^20,28,31–33^. Additionally, health services may have a limited epidemiological impact because they reach only a small proportion of individuals in need of treatment, a phenomenon often referred to as the "clinical iceberg"^34^. In the context of periodontitis, many cases may remain undiagnosed or asymptomatic, thereby failing to generate demand for care. Periodontal probing, considered the gold standard technique for diagnosing periodontitis^35^, is not consistently performed in routine dental examinations. The influence of socioeconomic factors on access to dental care in the development of periodontal disease reflects a pattern similar to that described for dental caries^36^.

An alternative explanation for the findings related to CEOs is that a potential effect of specialized services may not be detectable due to challenges in service work processes. For instance, it has been reported that most CEOs experience difficulties in meeting production targets^37–39^, whereas large municipalities, despite having lower PHC-OH, are more likely to achieve such targets in periodontics. One hypothesis for the lack of synergy between PHC-OH and CEOs is the presence of shortcomings in the articulation of the care network, particularly in the regulation and management of referral and counter-referral systems. In practice, the effectiveness of CEOs depends on the existence of a regulated, integrated, and efficient care flow, which remains a significant challenge in many settings. Previous evidence indicates that absenteeism rates are high (30%), being 1.22 times higher in periodontics referral queues^40^. Insufficient system coordination may lead to inappropriate utilization or create barriers to access, thereby compromising case resolution and contributing to the progression of periodontal conditions.

These care networks face multiple challenges, ranging from initial access to the provision of specialized services^41,42^. Referral practices vary across Brazilian municipalities^43^; in some settings, walk-in appointments are available^44^, whereas municipalities with more structured primary care tend to offer a greater supply of specialized services^45,46^. In this context, it is essential that managers and health professionals adopt appropriate implementation strategies, including structured training, internal facilitation, and continuous monitoring of periodontal referral indicators, in order to promote integrated care pathways, reduce absenteeism in CEOs, and ensure the appropriate use of the care network. Such an approach may enable the identification of these barriers to be translated into opportunities for learning and continuous improvement, thereby enhancing the effectiveness and comprehensiveness of care within periodontal care pathways.

In conclusion, greater coverage of primary dental care appears to be independently associated with a lower prevalence of periodontal disease in small- and medium-sized municipalities; however, the presence of specialized care demonstrated a weak and non-significant association. Health managers should consider these findings when planning the expansion of oral health teams in PHC as well as in specialized services, with the aim of establishing care networks capable of achieving universal and comprehensive care. Further studies are warranted to better understand the barriers within referral systems and to assess the effectiveness of PHC.
