# Supplementary material for: Relation between primary and secondary health care coverage with periodontal disease in Brazil: a multilevel study
Source: Rev Bras Epidemiol. 2026 Jun 12;29(Suppl 1):e260008supl1. doi: 10.1590/1980-549720260008.supl.1 (PMC13268397; doi:10.1590/1980-549720260008.supl.1)
Supplement: Tabela Suplementar [file 1980-5497-rbepid-29-suppl1-e260008supl1-supp01.pdf]

Tabela S1 - *Razão de Chances (OR) bruta e ajustada com intervalo de confiança 95% (IC95%) de ter ao menos um dente com periodontite (Escore CPI > 3mm bolsa) em adultos e idosos, SBBrasil 2023.*

		Modelo Bruto		Modelo Ajustado 1		Modelo Ajustado 2		Modelo Ajustado 3		Modelo Ajustado 4	
		OR	(IC95%)	OR	(IC95%)	OR	(IC95%)	OR	(IC95%)	OR	(IC95%)
Variáveis Municipais											
Presença de CEO	Nenhum	1				1		1		1	
	1 ou mais	1,39	(0,98 - 1,98)			1,42	(1 - 2,03)	1,08	(0,72 - 1,63)	1,14	(0,78 - 1,67)
Cobertura de Serviços de Atenção Primária em Saúde Bucal	<50%	1		1				1		1	
	50-74%	0,88	(0,6 - 1,31)	0,89	(0,6 - 1,33)			0,89	(0,61 - 1,29)	0,87	(0,6 - 1,27)
	75-99%	0,82	(0,52 - 1,29)	0,84	(0,53 - 1,32)			0,86	(0,54 - 1,38)	0,84	(0,53 - 1,32)
	100%	0,50	(0,32 - 0,78)	0,49	(0,31 - 0,76)			0,52	(0,31 - 0,89)	0,52	(0,31 - 0,87)
Encaminhamentos para Tratamento Periodontal / raspagem supra+subgengival	1º Tercil (menor)	1						1			
	2º Tercil (intermediário)	1,17	(0,81 - 1,71)					1,10	(0,74 - 1,64)		
	3º Tercil (maior)	1,07	(0,74 - 1,55)					1,03	(0,72 - 1,47		
Produto Interno Bruto Per capita	1º Tercil (PIB menor)	1						1		1	
	2º Tercil (PIB intermediário)	0,90	(0,63 - 1,29)					0,76	(0,51 - 1,14)	0,76	(0,51 - 1,13)
	3º Tercil (PIB maior)	1,33	(0,94 - 1,9)					1,16	(0,8 - 1,69)	1,11	(0,77 - 1,62)
Variáveis Individuais											
Sexo	Masculino	1		1		1		1		1	
	Feminino	1,50	(1,33 - 1,69)	1,40	(1,25 - 1,56)	1,41	(1,26 - 1,57)	1,41	(1,26 - 1,57)	1,45	(1,29 - 1,62)
Idade	35 a 44 anos	1		1		1		1		1	
	65 a 74 anos	1,43	(1,22 - 1,66)	1,13	(0,96 - 1,35)	1,14	(0,96 - 1,35)	1,13	(0,95 - 1,34)	1,14	(0,96 - 1,35)
Escolaridade	Fundamental Incompleto	1		1		1		1		1	
	Fundamental Completo	0,81	(0,67 - 0,98)	0,86	(0,71 - 1,05)	0,87	(0,72 - 1,06)	0,87	(0,71 - 1,05)	0,84	(0,69 - 1,02)
	Ensino Médio Completo	0,59	(0,5 - 0,69)	0,70	(0,59 - 0,84)	0,71	(0,59 - 0,84)	0,70	(0,59 - 0,84)	0,63	(0,52 - 0,76)
	Superior Completo	0,33	(0,22 - 0,5)	0,42	(0,28 - 0,62)	0,42	(0,28 - 0,62)	0,41	(0,28 - 0,61)	0,35	(0,23 - 0,54)
Tempo desde ultima visita ao dentista	Menos de um ano	1		1		1		1			
	1 a 2 anos	0,99	(0,8 - 1,23)	0,96	(0,77 - 1,19)	0,96	(0,77 - 1,19)	0,96	(0,78 - 1,19)		
	2 a 3 anos	1,28	(1,01 - 1,61)	1,17	(0,92 - 1,47)	1,17	(0,93 - 1,48)	1,17	(0,93 - 1,48)		
	>3 anos	1,55	(1,28 - 1,88)	1,29	(1,06 - 1,57)	1,29	(1,06 - 1,57)	1,29	(1,06 - 1,57)		
Motivo de última visita	Revisão/Manutenção	1		1		1		1			
	Dor/Exodontia	1,81	(1,46 - 2,23)	1,59	(1,31 - 1,93)	1,59	(1,31 - 1,92)	1,60	(1,32 - 1,93)		

	Tratamento	1,34	(1,1 - 1,62)	1,26	(1,05 - 1,51)	1,25	(1,04 - 1,5)	1,26	(1,05 - 1,51)		
Amostra	Indivíduos	6386		6309		6309		6309		6309	
	Municípios	325		325		325		325		325	
Ajuste do Modelo	BIC	6151		6019		6010		6059		6056	
	VPC	17,1%		16,2%		16,1%		13,9%		14,4%	
	Variância 2º nível	0,68		0,64		0,63		0,53		0,5530739	

Tabela S2 *- Prevalência ponderada de periodontite (CPI>3mm) em adultos e idosos usuários de serviços públicos estratificadas pela presença de Centro de Especialidade Odontológica (CEO) no Brasil 2023.*

	Presença de CEO no município			
	Nenhum		1 ou mais	
	% Periodontite	P-value	% Periodontite	P-value
Total	11,9		19,4	
**Variáveis Contextuais (Municipal)**				
Cobertura de Serviços de Atenção Primária em Saúde Bucal				
<50%	13,5	0,01	20,3	0,26
50-74%	25,6		19,9	
75-99%	8,4		22,4	
100%	6,1		9,9	
Encaminhamentos para Tratamento Periodontal / raspagem supra+subgengival				
1o Tercil (menor)	12,2	0,59	18,5	0,81
2o Tercil (intermediário)	12,7		20,9	
3o Tercil (maior)	5,8		18,4	
Produto Interno Bruto Per capita				
1o Tercil (PIB menor)	11,8	0,40	15,6	0,10
2o Tercil (PIB intermediário)	15,6		16,6	
3o Tercil (PIB maior)	7,3		22,8	
Porte Populacional (Habitantes)				
Até 100 mil	12,0	0,92	16,3	0,64
100 – 500 mil	11,5		18,5	
500 mil - 1 milhão			19,8	
>1 milhão			23,1	
**Variáveis Individuais**				
Sexo				
Masculino	22,5	0,39	13,9	0,14
Feminino	18,0		10,7	
Idade				
35 a 44 anos	17,7	0,05	10,7	0,03
65 a 74 anos	26,6		18,4	
Renda Equivalente				
<=1/2 Salário Mínimo	22,6	0,03	5,3	0,14
1/2-1 Salário Mínimo	17,9		17,8	
1-2 Salário Mínimo	19,0		13,5	
=>2 Salário Mínimo	8,3		19,9	
Escolaridade				
Fundamental Incompleto	30,2	0,29	13,7	<0.01
Fundamental Completo	19,4		13,5	
Ensino Médio Completo	13,8		11,1	
Superior Completo	7,5		2,6	
Tempo desde última visita ao dentista				
Menos de um ano	14,6	0,55	10,0	<0.01
1 a 2 anos	19,6		13,1	
2 a 3 anos	21,9		16,7	
>3 anos	31,8		13,1	
Motivo de última visita				
Revisão/Manutenção	12,5	0,59	10,6	<0.01
Dor/Exodontia	25,4		13,9	
Tratamento	18,4		11,0	

Tabela S3 - *Prevalência ponderada de periodontite (CPI>3mm) em adultos e idosos usuários de serviços públicos estratificadas pela cobertura de serviços de atenção primária em saúde bucal no Brasil 2023.*

	Cobertura de Serviços de Atenção Primária em Saúde Bucal							
	<50%		50-74%		75-99%		100,00%	
	% Periodontite	P-value	% Periodontite	P-value	% Periodontite	P-value	% Periodontite	P-value
Total	19,3		21,1		18,1		7,7	
**Variáveis Contextuais (municipais)**								
**Presença de CEO**								
Nenhum	13,5	0,35	25,6	0,44	8,4	0,04	6,1	0,22
1 ou mais	20,3		19,9		22,4		9,9	
**Encaminhamentos para Tratamento Periodontal / raspagem supra+subgengival**								
1o Tercil (menor)	15,1	0,51	25,4	0,41	12,2	0,04	8,2	0,46
2o Tercil (intermediário)	21,1		17,0		44,5		10,4	
3o Tercil (maior)	23,0		19,1		16,6		5,1	
**Produto Interno Bruto Per capita**								
1o Tercil (PIB menor)	18,1	0,73	18,0	0,66	18,0	0,38	7,5	0,58
2o Tercil (PIB intermediário)	16,2		22,7		11,7		12,4	
3o Tercil (PIB maior)	20,7		23,3		26,5		5,9	
**Porte Populacional (Habitantes)**								
Até 100 mil	18,2	0,50	25,1	0,40	19,5	0,23	6,6	<0.01
100 – 500 mil	15,6		19,6		11,6		25,7	
500 mil - 1 milhão	20,3		12,5		23,9		-	
>1 milhão	23,2		28,6		-		-	
**Variáveis Individuais**								
**Sexo**								
Masculino	22,5	0,26	21,0	0,97	21,2	0,41	10,7	0,10
Feminino	17,7		21,2		17,0		6,3	
**Idade**								
35 a 44 anos	16,7	0,02	21,2	0,93	16,3	0,07	6,6	0,03
65 a 74 anos	29,6		20,6		27,0		12,9	
**Renda Equivalente**								
<=1/2 Salário Mínimo	22,2	0,43	22,5	0,52	17,8	0,82	3,5	0,10
1/2-1 Salário Mínimo	18,3		30,1		14,1		9,9	
1-2 Salário Mínimo	18,3		21,9		15,6		9,5	
=>2 Salário Mínimo	9,3		12,6		9,1		1,1	
**Escolaridade**								
Fundamental Incompleto	31,5	<0.01	25,6	0,25	22,3	0,57	11,4	0,06
Fundamental Completo	18,5		26,4		13,3		10,2	
Ensino Médio Completo	12,3		19,3		18,1		4,0	
Superior Completo	8,2		3,4		12,9		1,6	
**Tempo desde última visita ao dentista**								
Menos de um ano	12,3	<0.01	22,1	0,61	8,9	<0.01	9,1	0,02

1 a 2 anos	23,9		17,6		17,8		1,9	
2 a 3 anos	27,3		14,0		18,6		5,0	
>3 anos	30,3		26,1		43,0		10,9	
Motivo de última visita								
Revisão/Manutenção	11,3	0,03	12,3	<0.01	20,4	0,70	5,6	0,17
Dor/Exodontia	23,1		33,1		18,5		10,4	
Tratamento	21,4		14,2		14,7		5,8	
