# Supplementary material for: Relation between primary and secondary health care coverage with periodontal disease in Brazil: a multilevel study
Source: Rev Bras Epidemiol. 2026 Jun 12;29(Suppl 1):e260008supl1. doi: 10.1590/1980-549720260008.supl.1 (PMC13268397; doi:10.1590/1980-549720260008.supl.1)
Supplement: Translation

# Translation: Relação entre cobertura de atenção primária e secundária com doença periodontal no Brasil: um estudo multinível

**DOI:** 10.1590/1980-549720260008.supl.1.2

**Authors:** Roger Keller Celeste, Leonardo Vilar Filgueiras, Joao Paulo Steffens, Stefany Duarte dos Anjos, Rafael Aiello Bomfim, Yuri Wanderley Cavalcanti, Alex Nogueira Haas

**Affiliations:** I Universidade Federal do Rio Grande do Sul – Porto Alegre (RS), Brasil.; II Universidade Federal do Paraná – Curitiba (PR), Brasil.; III Universidade Federal do Mato Grosso do Sul – Campo Grande (MS), Brasil.; IV Universidade Federal da Paraíba – João Pessoa (PB), Brasil.

**Keywords:** Serviços de saúde bucal, Atenção secundária, Atenção primária à saúde, Periodontite, Especialidades odontológicas, Avaliação de serviços de saúde

## Abstract

**Objetivo:**

Avaliar os efeitos independentes e conjuntos da presença de Centros de Especialidades Odontológicas (CEOs) e cobertura de atenção primária em saúde na Doença Periodontal (DP) entre adultos e idosos no Brasil.

**Métodos:**

Dados do inquérito SB Brasil 2023 foram obtidos para 7.851 indivíduos que utilizaram serviços públicos. Variáveis contextuais foram obtidas de sistemas de informação em saúde e do Instituto Brasileiro de Geografia e Estatística (IBGE). A variável de desfecho foi DP, definida como ter >1 dente com profundidade de sondagem >3 mm. As principais exposições foram a presença de CEOs e a cobertura da Atenção Primária em Saúde Bucal (eSB APS). Análises de regressão logística multinível foram realizadas.

**Resultados:**

A prevalência ponderada de DP foi de 17,4%; e, respectivamente, 19,4 e 11,9% (p=0,03) em municípios com e sem CEO. Municípios com cobertura populacional de eSB APS<50%, 50–74%, 75–99% e 100% apresentaram prevalências, respectivamente, de 19,3, 21,1, 18,1 e 7,7% (p=0,01). Indivíduos em municípios com CEOs apresentaram maior chance de desenvolver DP [OR 1,12 (IC95% 0,75–1,68)]. Indivíduos em municípios com 100% de cobertura de eSB APS apresentaram OR 0,57 (IC95% 0,29–1,12) menos chances de DP em comparação aos municípios com <50% na presença de CEO. Na ausência dos CEOs, o respectivo OR foi 0,43 (IC95% 0,19–97), mas a interação CEO e eSB APS não foi significativa (p=0,90).

**Conclusão:**

Maior cobertura de atenção primária em saúde bucal parece estar independentemente associada a menor prevalência de DP em cidades de pequeno e médio porte. Estudos futuros devem investigar as barreiras no sistema de encaminhamento.

## INTRODUÇÃO

As doenças periodontais mais prevalentes, gengivite e periodontite, são caracterizadas por um processo infecto-inflamatório que, se não tratado, pode levar à disfunção mastigatória pela destruição das estruturas de suporte dos dentes^1,2^. Além do impacto na função mastigatória e na estética, a periodontite está associada a repercussões sistêmicas relevantes, incluindo maior risco de doenças cardiovasculares, diabetes e complicações gestacionais, o que reforça sua importância como problema de saúde pública^3^. Ademais, ela afeta desigualmente pessoas de diferentes estratos sociais^4^ e acarreta forte impacto na qualidade de vida das pessoas^5^.

Clinicamente, a periodontite é definida pela presença de Perda de Inserção Clínica (PIC) e/ou perda óssea radiográfica. Outros critérios clínicos inflamatórios, como a Profundidade de Sondagem (PS) e o Sangramento à Sondagem (SS), podem complementar o diagnóstico clínico^6^. Em nível epidemiológico, o Índice Comunitário Periodontal (CPI) vem sendo preconizado pela Organização Mundial de Saúde (OMS) por sua facilidade de aplicação em grandes amostras, mas com a limitação de examinar parcialmente a dentição em apenas seis dentes-índice e por não avaliar PIC. Aproximadamente 12% da população global é afetada pela doença em seu estado mais severo^7^, e pelo menos 80% dos adolescentes apresentam sinais de gengivite^8,9^. No Brasil, dados da Pesquisa Nacional de Saúde Bucal 2023 (SB Brasil 2023)^10^ indicam que 13,4% dos adultos na faixa etária de 35 a 44 anos apresentam bolsas periodontais, correspondendo ao grupo etário com maior prevalência da doença.

Aproximadamente 80% das Unidades Básicas de Saúde (UBS) contam com a presença de cirurgiões-dentistas em suas equipes^11^. Entretanto, apesar de avanços significativos no financiamento e ampliação da rede de atenção em saúde bucal, fruto da Política Nacional de Saúde Bucal^12^, ainda permanecem desafios de diversas naturezas, como organização da demanda e regionalização de atenção em rede^13,14^. O uso de serviços e as desigualdades no acesso, em geral, estão relacionados a fatores socioeconômicos e organizacionais, mas, embora o uso tenha aumentado, as desigualdades no uso de serviços odontológicos não têm reduzido^15^. Além disso, há dificuldades no direcionamento de recursos e políticas públicas de saúde com base nas necessidades das pessoas, o que, por sua vez, dificulta uma distribuição apropriada na utilização dos serviços de saúde bucal^16,17^.

Há pouca evidência sobre os efeitos do uso dos serviços de atenção primária na saúde bucal e sobre o papel da atenção secundária no cuidado odontológico^18–20^. Segundo uma revisão sistemática recente, há estudos que mostram um impacto limitado do uso de serviços na atenção primária na saúde periodontal, sendo o aumento de cobertura associado a uma menor prevalência de doença periodontal^21^. No entanto, a maioria dos estudos não aborda a atenção especializada em odontologia, que no Brasil teve expansão por meio dos Centros de Especialidades Odontológicas (CEOs).

A presença dos CEOs parece estar associada a uma menor prevalência de doença periodontal entre adultos, e o seu efeito é parcialmente mediado pelo uso de serviços^19^. Assim, uma ampliação do acesso pode aumentar o uso dos serviços e talvez melhorar a saúde em nível populacional. Historicamente, há maior investimento na atenção primária em saúde bucal, porém, recentemente os serviços especializados, incluindo periodontia, também foram expandidos por meio do financiamento de CEO no Brasil^12,22^. Os resultados dessa política mostraram que isso tem levado a um maior uso dos serviços especializados. No entanto, o impacto epidemiológico ainda não está bem nítido.

A ampliação do acesso aos serviços especializados pode aumentar a resolutividade dos serviços de Atenção Primária à Saúde (APS) e, consequentemente, alterar o quadro epidemiológico. Por exemplo, foi descrito que os CEOs parecem reduzir o percentual de exodontia em municípios com maior cobertura de equipes de saúde bucal na Estratégia de Saúde da Família^23^. A resolutividade da APS pode ser aumentada, mantendo a cobertura constante, se os casos que demandam maior densidade tecnológica forem encaminhados para pontos da rede capazes de resolver tais problemas. Com isso, as equipes de APS focariam na demanda de acordo com suas capacidades. A hipótese do presente trabalho é de que as equipes de APS teriam maior capacidade de melhorar o quadro epidemiológico em municípios com CEOs. Assim, os objetivos são avaliar os efeitos independentes e conjuntos da presença de Centros de Especialidades Odontológicas e cobertura de atenção primária em saúde na doença periodontal entre adultos e idosos no Brasil.

## MÉTODOS

Trata-se de um estudo transversal que reuniu dados de diversas fontes. As variáveis individuais foram obtidas do SB Brasil de 2023^24^. Variáveis contextuais foram coletadas dos Sistemas de Informação em Saúde (SIS) de todos os 430 municípios participantes do SB Brasil. A amostra original totalizou 18.766 indivíduos das faixas etárias de 35 a 44 anos e 65 a 74 anos. Este estudo concentrou-se em indivíduos que utilizaram serviços públicos em sua última consulta, resultando em 7.851 indivíduos elegíveis. Esse critério foi utilizado porque é improvável que os serviços públicos afetem os usuários de outros tipos de serviço.

### Variável de desfecho

A variável de desfecho foi presença de doença periodontal (dicotômica: 0=não, 1=sim) medida em nível individual. Seis dentes índices, um em cada sextante, foram examinados através da aplicação do CPI utilizando a sonda da OMS. Apenas sextantes com 2 ou mais dentes sem indicação de exodontia foram avaliados. Cada sextante recebeu uma pontuação de acordo com a presença (códigos 3 e 4) ou não (códigos 0, 1 e 2) de PS>3 mm. A pontuação mais alta entre todos os sextantes foi utilizada para representar o indivíduo. Doença periodontal foi definida como ≥1 sextante com PS>3 mm (códigos 3 ou 4 do CPI).

### Variáveis de exposição

Todas as variáveis consideradas de exposição são em nível municipal. As principais variáveis de exposições foram presença de CEO (nenhum, 1 ou mais) e cobertura de saúde bucal na atenção primária (≤50, 50–74, 75–99 ou 100%). Em relação à variável presença de CEO, tal dicotomização justifica-se pelo pequeno número de municípios com múltiplos CEOs na amostra (45% nenhum, 41% com 1, 7% com 2, e 7% com mais de 2 centros). Já para a variável cobertura de saúde bucal na atenção primária, a categorização foi baseada na sua distribuição evitando a dicotomização e considerando que 33% dos municípios da amostra possuem 100% de cobertura e, ainda, que essa categoria inclui 10,8% dos indivíduos da amostra do SB Brasil. Assim, as categorias de cobertura (<50, 50–75, 75–99 e 100%) foram criadas considerando concomitantemente o número de municípios e de indivíduos em cada categoria, de forma a manter um equilíbrio de tamanho amostral de indivíduos e municípios.

O número de CEOs presentes em cada cidade foi obtido via informações disponibilizadas pelo Ministério da Saúde (Coordenação Geral de Saúde Bucal), disponível em: https://www.gov.br/saude/pt-br/composicao/saps/brasil-sorridente/cidades-atendidas/ceo. As informações foram extraídas do sistema e-gestor do Ministério da Saúde, utilizando-se o mês de dezembro de 2022 como referência, e estão disponíveis em: https://relatorioaps.saude.gov.br/cobertura/saude-bucal/v2. Para o cálculo da cobertura foi levada em conta a cobertura de saúde bucal total, considerando unidades de saúde bucal na Estratégia de Saúde da Família (equipes e-SFSB) e, também, aquelas na Atenção Primária à Saúde (eSB APS).

Além dessas variáveis, o número de encaminhamentos para tratamento periodontal foi obtido do Sistema de Informação da Atenção Básica (Sisab), bem como o número total de procedimentos de intervenção periodontal (total de raspagens supra e subgengivais) na atenção primária para o período entre janeiro/2022 e dezembro/2023. Foi então criada uma taxa, dividindo-se os encaminhamentos pelo total de procedimentos de raspagem.

### Variáveis de ajuste

Utilizando-se um modelo teórico-operacional desenvolvido para este estudo (Figura 1), as associações principais foram ajustadas por potenciais fatores de confusão. O modelo também permitiu identificar potenciais fatores de mediação da relação entre as principais exposições e o desfecho. As covariáveis utilizadas no modelo foram identificadas com base em estudos prévios descritos na introdução do presente estudo e agrupadas em individuais e contextuais de confusão ou mediadoras. Diferentemente do modelo teórico-operacional, a variável de renda domiciliar não foi utilizada devido ao alto percentual de valores faltantes (26,5%, n=2.084).

**Figura 1:** Modelo teórico-operacional de análise.

As variáveis individuais para controle composicional, obtidas do inquérito SB Brasil, foram: sexo (masculino/feminino), idade (anos), escolaridade (fundamental incompleto, fundamental completo, ensino médio completo e superior completo), tempo desde a última consulta ao dentista (<1 ano, 1 a 2 anos, 2 a 3 anos, >3 anos) e o motivo da consulta (revisão/manutenção, dor/exodontia, tratamento). As variáveis em nível municipal, como porte populacional e Produto Interno Bruto (PIB), em reais, foram obtidas de dados do Censo de 2022 disponíveis no site do Instituto Brasileiro de Geografia e Estatística (IBGE). O PIB foi dividido pela população municipal e categorizado em tercis, sendo o primeiro tercil com menor valor monetário. O porte populacional foi classificado em quatro categorias (até 100 mil habitantes, 100–500 mil habitantes, 500 mil-1 milhão de habitantes e >1 milhão de habitantes).

### Análise estatística

Os dados descritivos foram apresentados usando pesos amostrais para estimar prevalências e médias representativas. Os pesos amostrais foram calculados com base nas frações amostrais de cada estágio amostral e calibrados para corrigir a não resposta. Foi realizada regressão logística multinível com intercepto aleatório para estimar o efeito ajustado das principais variáveis municipais na doença periodontal. Os indivíduos foram aninhados em seus respectivos municípios, as unidades de segundo nível. A função de ligação logit foi utilizada, e seus valores β exponenciados são Razões de Chances (OR). Por fim, o Coeficiente de Partição da Variância (VPC) foi calculado utilizando o Método D proposto por Goldstein et al.^25^

Inicialmente, modelos com termos de interação foram ajustados para os fatores de confusão, conforme o modelo teórico-operacional (Figura 1), utilizando uma estrutura de análise hierárquica. Após, quatro modelos de regressão múltipla sem interações foram ajustados, em adição aos modelos dos efeitos não ajustados. O primeiro modelo (M1) ajustou a associação da cobertura de atenção primária por fatores de confusão com variáveis em nível individual. O segundo modelo (M2) ajustou a associação da presença de CEOs por fatores de confusão com variáveis em nível individual. O terceiro modelo (M3) incluiu todas as variáveis individuais e contextuais, enquanto o quarto modelo (M4) excluiu as variáveis de uso de serviços de saúde (potenciais mediadores). Análise adicional com tabelas bivariadas para variáveis categóricas identificou forte colinearidade (caselas zeradas ou com pequena amostra) da presença de CEOs e cobertura de eSB APS com porte populacional, sendo apresentada análise estratificada. O ajuste de cada modelo foi avaliado usando o critério de informação bayesiano. Todas as análises foram realizadas usando o software estatístico *Stata* 19.0.

#### Declaração de disponibilidade de dados:

Dados disponíveis mediante solicitação. Todo o conjunto de dados que dá suporte aos resultados deste estudo está disponível mediante solicitação ao Ministério da Saúde (https://www.gov.br/saude/pt-br/composicao/saps/brasil-sorridente/sb-brasil/dados)

## RESULTADOS

A amostra do estudo totalizou 7.851 indivíduos elegíveis. Desse total, 18,7% (n=1.465) foram perdas, das quais 16,3% (n=1.273) eram edêntulos e 2,4% (n=192), dados faltantes para doença periodontal, sendo a amostra analítica final composta de 6.386 indivíduos. O coeficiente de partição de variância identificou que 17,1% da variabilidade total é fruto de diferenças entre municípios; e mesmo após ajuste por confundimento composicional permaneceu em torno de 14% (Tabela Suplementar 1).

A prevalência total de doença periodontal foi de 17,4% (Tabela 1). A prevalência foi significativamente maior em municípios com presença de CEO (19,4%) do que na ausência dos centros (11,9%). Maiores prevalências também foram observadas em municípios com menores coberturas de serviços de eSB APS. Não houve diferença significativa na prevalência de DP entre categorias de encaminhamentos para tratamento periodontal, mas maiores prevalências foram observadas em municípios com maiores PIBs e portes populacionais, apesar de não terem sido estatisticamente significantes (p<0,05). Em relação a variáveis individuais, não foram observadas diferenças entre os sexos e as faixas de renda. No entanto, idosos tiveram maior prevalência do que adultos, assim como maior prevalência foi observada para pessoas com menor escolaridade. Em relação ao tempo desde a última visita, indivíduos que levaram 2 a 3 anos para serem atendidos possuíam maior prevalência de doença periodontal. Da mesma forma, maior prevalência foi observada para pessoas que procuraram atendimento de urgência em comparação àquelas que procuraram atendimento para manutenção da saúde.

**Tabela 1:** Prevalência ponderada de doença periodontal (CPI≥3) em adultos e idosos usuários de serviços públicos de acordo com variáveis contextuais municipais e individuais no Brasil, 2023.

		Total		Prevalência ponderada		
		%	n	%	Casos	Valor de p
Total		100,0	6.386	17,4	1.254	
Variáveis contextuais (municipal)						
Presença de CEO						
	Nenhum	26,7	827	11,9	827	0,03
	1 ou mais	73,3	5.559	19,4	5.559	
Cobertura de serviços de atenção primária em saúde bucal						
	<50%	46,7	4.185	19,3	3.487	0,01
	50–74%	21,2	1.381	21,1	1.146	
	75–99%	12,8	1.385	18,1	1.092	
	100%	19,4	900	7,7	661	
Encaminhamentos para tratamento periodontal/raspagem supra+subgengival						
	1° tercil (menor)	44,4	1.991	15,3	1.991	0,43
	2° tercil (intermediário)	30,3	2.192	20,3	2.192	
	3° tercil (maior)	25,4	2.203	17,6	2.203	
PIB per capita						
	1° tercil (PIB menor)	41,0	1.999	14,1	1.999	0,09
	2° tercil (PIB intermediário)	18,6	2.252	16,2	2.158	
	3° tercil (PIB maior)	40,5	2.135	21,0	2.229	
Porte populacional (habitantes)						
	Até 100 mil	34,9	1.059	13,8	1.074	0,18
	100–500 mil	36,9	1.471	17,2	1.158	
	500 mil–1 milhão	10,0	1.713	19,7	1.808	
	> 1 milhão	18,2	2.143	23,0	2.346	
Variáveis individuais						
Sexo						
	Masculino	32,5	2.065	19,9	2.065	0,13
	Feminino	67,5	4.321	16,2	4.321	
Idade (anos)						
	35 a 44	81,8	4.051	84,3	4.051	<0,01
	65 a 74	18,2	2.335	75,3	2.335	
Renda equivalente (salário-mínimo)						
	≤1/2	22,7	927	16,0	927	0,34
	1/2–1	37,7	1.917	17,9	1.917	
	1–2	30,7	1.467	17,8	1.467	
	≥2	8,9	401	9,5	401	
Escolaridade						
	Fundamental incompleto	28,5	2.082	24,6	2.082	<0,01
	Fundamental completo	23,1	1.360	18,0	1.360	
	Ensino médio completo	41,3	2.415	13,2	2.415	
	Superior completo	7,1	465	6,3	465	
Tempo desde última visita ao dentista (anos)						
	Menos de um	51,9	2.935	13,4	2.935	<0,01
	1 a 2	19,3	1.271	17,8	1.271	
	2 a 3	12,6	911	20,6	911	
	>3	16,3	1.269	27,2	1.269	
Motivo de última visita						
	Revisão/manutenção	30,9	1.794	11,9	1.794	<0,01
	Dor/exodontia	38,6	2.739	22,4	2.739	
	Tratamento	30,5	1.840	16,4	1.840	

CEO: Centros de Especialidades Odontológicas; PIB:Produto Interno Bruto.

Descreveu-se a prevalência de doença periodontal pela presença de CEO (Tabela Suplementar 2) e pela cobertura de eSB na APS (Tabela Suplementar 3). Verificou-se que a cobertura de eSB APS teve associação mais forte com DP somente em municípios sem CEO. As demais variáveis municipais não tiveram associação significativa em nenhum estrato de presença ou ausência de CEOs. Independentemente da cobertura de APS, não houve diferença na prevalência de doença periodontal entre as categorias das demais variáveis municipais, embora o padrão de associação tenha sido aproximadamente o mesmo. Para as variáveis individuais, observaram-se associações significativas na prevalência de doença periodontal entre algumas categorias de idade, escolaridade, tempo desde a última visita e motivo da visita odontológica.

Na Tabela 2 os modelos com interação entre CEO e eSB APS são apresentados, sendo não significativas tanto no modelo bruto (p=0,88) como no ajustado (p=0,90). Em municípios sem a presença de CEO, 100% de cobertura de eSB APS esteve associada a menor chance de doença periodontal em 57% (OR 0,43 IC95% 0,19–0,97). Para as demais estimativas de razão de chance os intervalos de confiança cruzam o valor 1. Foram testadas outras cinco interações com a presença de CEOs (CEO#porte populacional, com p=0,75; CEO#encaminhamentos para periodontia, com p=0,25; CEO#PIB, com p=0,19; CEO#tempo desde última visita ao dentista, com p=0,51; e CEO#motivo da última visita ao dentista, com p=0,35) e para cobertura de eSB APS (APS#porte populacional, com p=0,11; APS#encaminhamentos para periodontia, com p=0,31; APS#PIB, com p=0,79; APS#tempo desde última visita ao dentista, com p=0,14; e APS#motivo da última visita ao dentista, com p=0,61).

**Tabela 2:** Razão de chances e intervalo de confiança 95% de ao menos um dente com escore Índice Comunitário Periodontal ≥3 para presença de Centro de Especialidade Odontológica segundo níveis de cobertura de atenção primária em saúde bucal em adultos e idosos, SB Brasil 2023.

Número de CEOs	Cobertura de serviços de atenção primária em saúde bucal	Modelo bruto		Modelo ajustado*	
		OR	(IC95%)	OR	(IC95%)
Nenhum	<50%	1		1	
	50–74%	0,98	(0,35–2,68)	0,88	(0,32–2,42)
	75–99%	0,84	(0,30–2,35)	0,68	(0,24–1,93)
	100%	0,46	(0,21–1,03)	0,43	(0,19–0,97)
Ao menos um	<50%	1		1	
	50–74%	0,86	(0,57–1,31)	0,82	(0,55–1,24)
	75–99%	0,82	(0,50–1,36)	0,90	(0,54–1,51)
	100%	0,63	(0,34–1,15)	0,57	(0,29–1,12)
Valores de p para interações CEO com cobertura APS		0,88		0,90	

OR: odds ratio; IC: intervalo de confiança; CEO: Centros de Especialidades Odontológicas; PIB: Produto Interno Bruto; APS: Atenção Primária em Saúde.

* ajustado por: sexo, idade, educação, tempo desde a última consulta, motivo da última consulta, PIB per capita, razão de encaminhamentos por raspagens na APS.

Considerando a colinearidade entre porte populacional com a presença de CEOs e eSB na APS, modelos estratificados são apresentados na Tabela 3. O efeito da cobertura total de eSB APS é significativo apenas em municípios com baixo porte populacional, e maior cobertura esteve associada a menor prevalência de DP (OR 0,41 IC95% 0,18–0,97). Em municípios de maior porte não foi possível avaliar associação com aumento de cobertura pela ausência de municípios com >500 mil habitantes com alta cobertura. Tal problema também aconteceu com CEOs, visto que todos os municípios de grande porte apresentavam CEOs. Modelos cheios reportando OR ajustando para porte populacional e para diferentes variáveis são apresentados na Tabela Suplementar 1. Destacando que a associação entre cobertura de 100% pela eSB APS e menor prevalência de DP se manteve (OR 52 IC95% 0,31–0,89).

**Tabela 3:** Razão de chances ajustada com intervalo de confiança 95% de ter ao menos um dente com Índice Comunitário Periodontal ≥3 em adultos e idosos estratificada por porte populacional, SB Brasil 2023.

		Modelo <100 mil hab.		Modelo 100–499 mil hab.		Modelo 500–999 mil hab.		Modelo >1 milhão hab.	
		OR	(IC95%)	OR	(IC95%)	OR	(IC95%)	OR	(IC95%)
Variáveis municipais									
CEO*									
	Nenhum	1		1		-	-	-	-
	1 ou mais	1,15	(0,50–2,66)	1,53	(0,63–3,70)	-	-	-	-
Cobertura de atenção primária em saúde bucal									
	<50%	1		1		1		1	
	50–74%	0,86	(0,31–2,39)	0,91	(0,42–1,97)	0,77	(0,40–1,48)	1,46	(1,18–1,80)
	75–99%	0,82	(0,32–2,10)	0,57	(0,24–1,33)	1,72	(0,25–11,6)	-	-
	100%	0,41	(0,18–0,97)	0,81	(0,30–2,15)	-	-	-	-
Tercis de encaminhamento†									
	T1 (menor)	1		1		1		1	
	T2 (intermediário)	1,10	(0,40–3,01)	0,64	(0,31–1,31)	2,02	(0,48–8,42)	1,34	(0,94–1,92)
	T3 (maior)	0,79	(0,33–1,91)	0,81	(0,43–1,55)	2,83	(0,67–12,0)	1,20	(0,81–1,78)
Tercis de PIB per capita‡									
	T1 PIB (menor)	1		1		1		1	
	T2 PIB (intermediário)	0,68	(0,32–1,48)	0,69	(0,30–1,58)	0,76	(0,12–4,86)	0,77	(0,40–1,48)
	T3 PIB (maior)	1,20	(0,57–2,55)	0,83	(0,39–1,79)	1,33	(0,19–9,16)	1,58	(0,81–3,08)
Amostra									
	Indivíduos	1.039		1.448		1.693		2.125	
	Municípios	189		97		24		13	
Ajuste de modelo									
	BIC	938,0		1.357,3		1.657,8		2.362,2	
	VPC	29,1%		17,5%		3,7%		2,1%	
	Variância 2° nível	1,35		0,70		0,13		0,07	

hab: habitantes; CEO: Centros de Especialidades Odontológicas; PIB: Produto Interno Bruto.

Nota: modelos ajustados por idade, sexo, educação, tempo desde a última visita ao dentista e motivo da última consulta.

* Presença de Centros de Especialidades Odontológicas no município;

† Encaminhamentos para tratamento periodontal/raspagem supra+subgengival na atenção primária;

‡ Produto Interno Bruto per capita;

§ Indicadores de ajuste do modelo.

## DISCUSSÃO

O presente estudo descreve três importantes resultados. Primeiro, não foi possível identificar uma sinergia entre a presença de CEOs e a cobertura de eSB APS. Há escassa evidência sobre tal achado, e nenhum estudo foi encontrado até o momento, embora seja teoricamente plausível^23^. Segundo, foi observada uma associação não significativa após ajustes entre a presença de CEOs e a prevalência de doença periodontal, confirmando um achado prévio com dados representativos do Brasil em 2010^19^. Por fim, um gradiente na associação entre maior cobertura de eSB APS e menor prevalência de doença periodontal foi confirmado, como previamente descrito^21,26–28^. O fato de todos os municípios com >500 mil habitantes possuírem CEO e nenhum apresentar cobertura de 100% da APS sugere que tais variáveis podem refletir características estruturais urbanas (e.g., desigualdades socioeconômicas, mobilidade urbana, sobrecarga assistencial), e talvez não um efeito causal direto dos serviços. Assim, tal associação só pode ser inferida para municípios com menos de 500 mil habitantes, dada a ausência de cobertura suficiente em municípios de maior porte. A variância residual atribuída ao nível municipal, na casa de 14%, significa que variáveis contextuais têm um papel importante a ser elucidado.

Este estudo teve limitações e pontos fortes. Uma limitação comum a todos os estudos observacionais é a impossibilidade de estabelecer uma relação temporal objetiva. Embora a cobertura de serviços seja uma característica municipal relativamente estável, a mobilidade urbana entre municípios de cobertura díspares pode ser considerável, introduzindo vieses. Além disso, este estudo não pôde controlar algumas variáveis de confusão, como tabagismo e diabetes. A periodontite é atualmente definida como uma doença inflamatória crônica multifatorial que tem diabetes e tabagismo como fatores de risco clássicos que podem, inclusive, alterar sua classificação em relação ao risco de progressão (grau)^6^. Mais recentemente, um corpo crescente de evidências tem demonstrado seu desenvolvimento como comorbidade ou multimorbidade de outras Doenças Crônicas não Transmissíveis (DCNTs), geralmente resultantes de fatores de risco comuns como o tabagismo^29^. Outro aspecto importante a ser destacado é a limitação do CPI em definir periodontite. A falta de informação sobre perda de inserção clínica em exame completo, que é o critério padrão-ouro para definir periodontite, pode gerar um viés de definição de doença nesta amostra do SB Brasil.

Os pontos fortes deste estudo incluíram dados de amostras representativas da população geral usando apenas usuários de serviços de saúde pública. O estudo utilizou informações oficiais de sistemas de informação em saúde. Além disso, o desfecho relacionado à profundidade de sondagem (bolsa periodontal) é relativamente sensível à necessidade de tratamento, diferentemente de perda de inserção, a qual reflete a história prévia da doença. Por outro lado, deve-se considerar que esta escolha metodológica pode ter resultado na inclusão de falsos positivos — indivíduos que tenham crescimento gengival por diferentes fatores — ou na exclusão de casos verdadeiros de doença periodontal — em fenótipos de progressão caracterizados pela perda progressiva de inserção sem formação concomitante de bolsa periodontal^30^. Finalmente, não foi possível estimar a extensão e a gravidade da doença periodontal devido a limitações metodológicas, visto que o inquérito não utiliza um protocolo de exame de todos os dentes.

O presente estudo não confirmou a associação entre acesso a serviços especializados odontológicos em nível municipal e doença periodontal. Tal ausência de efeito pode ter várias hipóteses explicativas. Uma explicação reside no fato de que o acesso aos serviços de saúde pode não ter real impacto no quadro epidemiológico. Estudos prévios indicam fraca ou ausente influência dos serviços odontológicos em relação a cárie dentária, cálculo dentário, periodontite e gengivite^20,28,31–33^. Além disso, os serviços de saúde podem ter impacto epidemiológico reduzido por cobrirem apenas uma pequena proporção daqueles que precisam de tratamento, caracterizando o "iceberg clínico"^34^. Em relação à periodontite, muitos casos podem não ser diagnosticados ou ser assintomáticos, não gerando demanda de tratamento. A sondagem periodontal, técnica padrão-ouro para se diagnosticar a periodontite^35^, nem sempre é rotina no exame odontológico. A importância dos fatores socioeconômicos nos cuidados dentários no desenvolvimento da doença periodontal reflete um padrão descrito para cárie dentária^36^.

Outra explicação para o achado acerca dos CEOs é que um real efeito de serviços especializados pode não ser detectável por dificuldades de processos de trabalho nos serviços. Por exemplo, é relatado que a maioria dos CEOs possui dificuldade de atingir as metas de produção^37–39^, sendo que municípios de grande porte, apesar de terem menor cobertura de eSB APS, são mais prováveis de atingirem tais metas em periodontia. Uma hipótese para explicar a ausência de sinergia entre os serviços da eSB APS e os CEOs podem ser falhas na articulação da rede de atenção, especialmente na regulação da gestão das filas de referência e contrarreferência. Na prática, a efetividade do CEO só se concretiza quando há um fluxo regulado, integrado e resolutivo que ainda é um grande desafio em muitos contextos. Um estudo prévio mostra que o absenteísmo é alto (30%), sendo 1,22 vezes maior na fila da periodontia^40^. Uma desarticulação no sistema pode resultar em uso inadequado ou criar barreiras de uso, comprometendo a resolutividade dos casos e contribuindo para o agravamento das condições periodontais.

Diversos são os desafios destas redes, desde o acesso de primeiro contato até a oferta de serviços especializados^41,42^. As formas de encaminhamento diferem entre os municípios brasileiros^43^; alguns ainda ofertam até o atendimento por livre demanda^44^, sendo que os municípios com melhor organização da atenção básica tendem a ter maior oferta especializada^45,46^. Assim, é fundamental que gestores e profissionais adotem estratégias de implementação adequadas, como treinamentos estruturados, facilitação interna e monitoramento contínuo de indicadores de encaminhamentos em periodontia, para promover fluxos integrados, reduzir o absenteísmo nos CEOs e garantir o uso adequado da rede de atenção. Essa abordagem permitiria transformar o reconhecimento dessas barreiras em oportunidades de aprendizagem e melhoria contínua, ampliando a resolutividade e a integralidade da atenção em fluxos de periodontia.

Concluindo, uma maior cobertura de cuidados odontológicos primários parece estar independentemente associada a uma menor prevalência de doença periodontal em cidades de pequeno e médio porte, porém, a presença de atenção especializada apresentou uma associação fraca e não significativa. Os gestores de saúde deveriam considerar tais questões na instalação de novas equipes de saúde bucal na atenção primária em saúde bem como na atenção especializada, criando uma rede de atenção capaz de atingir universalidade com integralidade da atenção. Futuros estudos são necessários para compreender os entraves no sistema de referenciamento e na resolubilidade de atenção primária em saúde.

## Material suplementar

Tabela Suplementar
